# Exploration of the residues modulating the catalytic features of human carbonic anhydrase XIII by a site-specific mutagenesis approach

**DOI:** 10.1080/14756366.2019.1653290

**Published:** 2019-08-20

**Authors:** Giuseppina De Simone, Anna Di Fiore, Emanuela Truppo, Emma Langella, Daniela Vullo, Claudiu T. Supuran, Simona Maria Monti

**Affiliations:** a Istituto di Biostrutture e Bioimmagini-CNR, Naples, Italy;; b Neurofarba Department, Università degli Studi di Firenze, Sezione di Scienze Farmaceutiche e Nutraceutiche, Sesto Fiorentino, Florence, Italy

**Keywords:** Cytosolic human carbonic anhydrases, catalytic activity, proton transfer, histidine cluster, site-specific mutagenesis

## Abstract

Carbonic anhydrases (CAs) are ubiquitous metallo-enzymes that catalyse the reversible hydration of carbon dioxide to bicarbonate and proton. In humans there are 15 isoforms among which only 12 are catalytically active. Since active human (h) CAs show different efficiency, the understanding of the molecular determinants affecting it is a matter of debate. Here we investigated, by a site-specific mutagenesis approach, residues modulating the catalytic features of one of the least investigated cytosolic isoform, i.e. hCA XIII. Results showed that residues assisting the formation of an ordered solvent network within the catalytic site as well as those forming a histidine cluster on the protein surface are important to guarantee an efficient proton transfer.

## Introduction

Human carbonic anhydrases (hCAs) are ubiquitous zinc-enzymes that catalyse the reversible hydration of carbon dioxide to bicarbonate and proton^1–3^. They exist in 15 isoforms, which are differently localised in cell. Among these, only 12 are catalytically active (CAs I–IV, VA, VB, VI, VII, IX, and XII–XIV), while the remaining 3 isoforms (CAs VIII, X, and XI) do not show enzymatic activity and are termed CA-related proteins (CARPs)^1–3^. Because of their involvement in a great variety of physiological and pathological processes^2^^,^^4–8^, hCAs have been extensively investigated both from a biochemical and structural point of view. In particular, structural studies showed that, independently from their subcellular localisation and in agreement with their high sequence homology, hCAs maintain a very similar fold, consisting of a central twisted β-sheet surrounded by several helices and additional β-strands. The active site is located in a large cavity which extends from the protein surface to the centre of the molecule, on the bottom of which the catalytic zinc ion is located being tetrahedrically coordinated by three conserved histidine residues and a water molecule/hydroxide ion^9–19^. The CA catalysed reaction follows a two-step mechanism described by Equations (1) and (2)^20–23^

(1) $${\text{EZn}}^{2+}-{\text{OH}}^{-}+{\text{ CO}}_{2}\;⇆\;{\text{EZn}}^{2+}-{\text{HCO}}_{3}^{-}\overset{{\text{H}}_{2}\text{O}}{⇆}{\text{EZn}}^{2+}-{\text{H}}_{2}\text{O }+{\text{ HCO}}_{3}^{-}$$

(2) $${\text{EZn}}^{\text{2}+}-{\text{H}}_{\text{2}}\text{O}+\text{B}\;⇆\;{\text{EZn}}^{\text{2}+}-{\text{OH}}^{-}+{\text{BH}}^{+}$$

In the first step, the Zn^2+^-bound hydroxide carries out a nucleophilic attack to CO_2_ with the formation of HCO_3_^–^. Since the binding of the latter ion to the zinc is rather labile, it is replaced by a water molecule leading to the catalytically inactive form of the enzyme (Equation (1)). The second step is the rate-limiting one and consists of the regeneration of the Zn^2+^-bound hydroxide species through a proton transfer reaction from the zinc-coordinated water molecule to the bulk solvent (Equation (2))^20–23^. In most of the human isoforms, a histidine residue positioned in the middle of the active site cavity, namely His64, assists this step by acting as a proton shuttle^24–28^.

**Figure 1.**
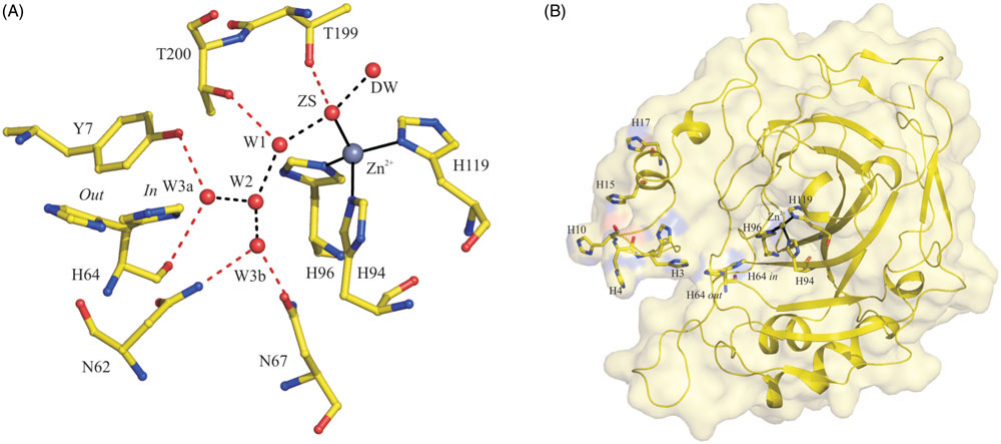
(A) View of hCA II active site showing the ordered water network which connects the Zn^2+^-bound solvent molecule (ZS) to the His64 proton shuttle residue. The solvent molecules are named as reported by Fisher et al. (PDB accession code 1TE3)^26^. Hydrogen bonds connecting the water molecules are coloured in black, whereas those between water molecules and protein residues are coloured in red. (B) Structure of hCA II showing the histidine residues which form a channel from the active site to protein surface. Zn^2+^ coordination is also depicted.

Kinetic studies on the different hCA isoforms revealed significant differences in their catalytic efficiency. Indeed, focusing the attention on the cytosolic isozymes, it has been observed that hCA II and hCA VII are very efficient catalysts (*K*_cat_/*K*_M_ values of 1.5 × 10^8^ M^−1^ s^−1^ and 8.3 × 10^7^ M^−1^ s^−1^ for hCA II and hCA VII, respectively), hCA I and hCA XIII possess medium efficiency (for hCA I *K*_cat_/*K*_M_ = 5.0 × 10^7^ M^−1^ s^−1^ and for hCA XIII *K*_cat_/*K*_M_ = 1.1 × 10^7^ M^−1^ s^−1^), while hCA III acts as a very poor catalyst (*K*_cat_/*K*_M_ = 3.0 × 10^5^ M^−1^ s^−1^)^2^. Although a large number of studies have been so far reported on these enzymes, the complete understanding of the molecular factors contributing to the observed differences in the catalytic efficiency is still a matter of debate. Most of the information so far available in this field derives from studies on hCA II, one of the most catalytically efficient isoforms. In particular, it has been suggested the important role of a well-ordered network of hydrogen bonded water molecules which connects the Zn^2+^-bound solvent molecule to His64 (Figure 1(A))^23^. These water molecules interact with several hydrophilic active-site residues, such as Tyr7, Asn62, Asn67, Thr199, and Thr200, which also contribute to the maintaining of an appropriate electrostatic environment for optimal proton transfer as shown by several mutagenesis, computational, structural, and kinetic studies^29–32^. Being these residues not strictly conserved in all hCA isoforms, it has been proposed that they could account for the diverse catalytic properties of this class of enzymes^30^^,^^33–35^. Also residues Ala65, Leu198, and Leu204 were recognised as important for the high catalytic efficiency of hCA II. In particular, the introduction of a bulky side chain in position 65 was demonstrated to induce a re-organisation of the solvent network as previously discussed^36^^,^^37^, whereas replacements of Leu198 and Leu204 were described to alter the position of His64 and thus the proton transfer^37^. Finally, an additional structural feature was proposed by Briganti et al., as responsible for hCA II highest catalytic efficiency^38^. This isoform has a cluster of histidines, namely His3, His4, His10, His15, and His17, which extends from the interior of the active site to its entrance and, finally, to the surface of the protein (Figure 1(B)). Authors hypothesised that this cluster could act as a sort of “channel” able to connect the His64 proton shuttle to the surface of the enzyme, thus facilitating the proton transfer step^38^. Additionally, it was also hypothesised that the histidine cluster could play a role in affecting the p*K*_a_ of His64^15^.

hCA XIII is one of the least investigated human cytosolic isoforms. It is expressed in the reproductive organs where it may control pH and ion balance regulation, ensuring appropriate fertilisation conditions^39^^,^^40^. Despite the high degree of sequence identity between hCA XIII and the very efficient hCA II (60%), this enzyme is one of the least efficient cytosolic isoforms second only to hCA III^2^^,^^41^. The crystallographic structure of hCA XIII was solved in 2009 by our group^18^^,^^42^, providing interesting hypotheses on the molecular determinants responsible for its catalytic properties. In particular, the absence of a well-defined histidine cluster was identified as the main factor determining the lower catalytic efficiency of this isoform with respect to hCA II^18^.

In order to investigate the role of amino acids in the active site which modulate the catalytic features of hCA XIII, a detailed structure-based comparison with hCA II active site was carried out highlighting the differences in residue composition. Diverging residues were replaced by site-specific mutagenesis and variants were expressed, purified, and kinetically tested. Our results reveal that the contribution to the enzymatic properties of each mutated residue is different, the catalytic efficiency being affected by the ordered solvent network and/or the histidine cluster.

## Materials and methods

### Cloning, expression, and purification of hCA XIII wild type and its variants

hCA XIII wild type was prepared as previously described^18^. hCA XIII L3H/S4H, S62N, S65A, R91I, and V200T variants were supplied by Blue Heron Biotechnology into the vector pUCminusMCS, all in frame for cloning in pGex-4T-3 vector (GE Healthcare). The sequences of the variants were confirmed by DNA sequencing. Expression and purification of the mutated enzymes were carried out as wild type isoform^18^. Sodium dodecyl sulphate-polyAcrylamide gel electrophoresis and liquid chromatography-electrospray ionization-mass spectrometry (LC-ESI-MS) experiments on purified variants confirmed their integrity and purity.

### Catalytic activity assays

All measurements were done according to Khalifah’s stopped flow method^43^. An Applied Photophysics stopped-flow instrument has been used for assaying the CA catalysed CO_2_ hydration activity. Phenol red (at a concentration of 0.2 mM) has been used as indicator, working at the absorbance maximum of 557 nm, with 20 mM Hepes as buffer (pH 7.4). About 20 mM NaClO_4_ were also added to the assay system for maintaining constant ionic strength. The initial rates of the CA-catalysed CO_2_ hydration reaction were followed for a period of 10–100 s. The CO_2_ concentrations ranged from 1.7 to 17 mM for the determination of the kinetic parameters and inhibition constants. The uncatalysed rates were determined in the same manner and subtracted from the total observed rates, as reported earlier^44–46^.

## Results and Discussion

**Figure 2.**
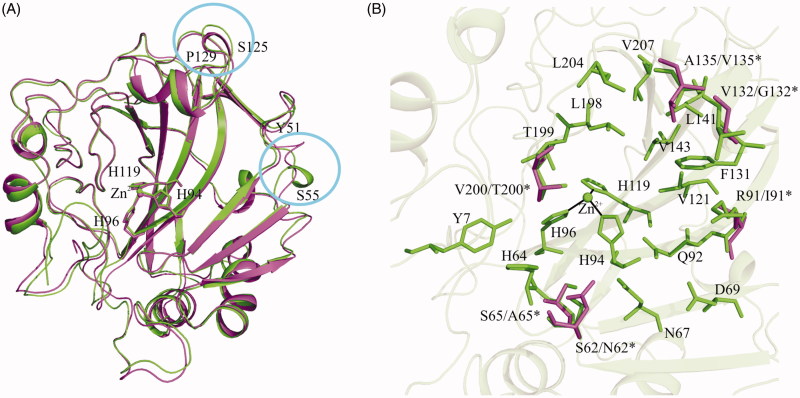
(A) Structural superposition of hCA XIII (green) and hCA II (magenta). The regions containing the main structural differences between the two enzymes are indicated with a circle (Ser125-Pro129 and Tyr51-Ser55). Zn^2+^ and its three histidine ligands are also represented. (B) Enlarged view of hCA XIII active site region. Diverging residues of hCA II are indicated by an asterisk and are coloured in magenta.

**Figure 3.**
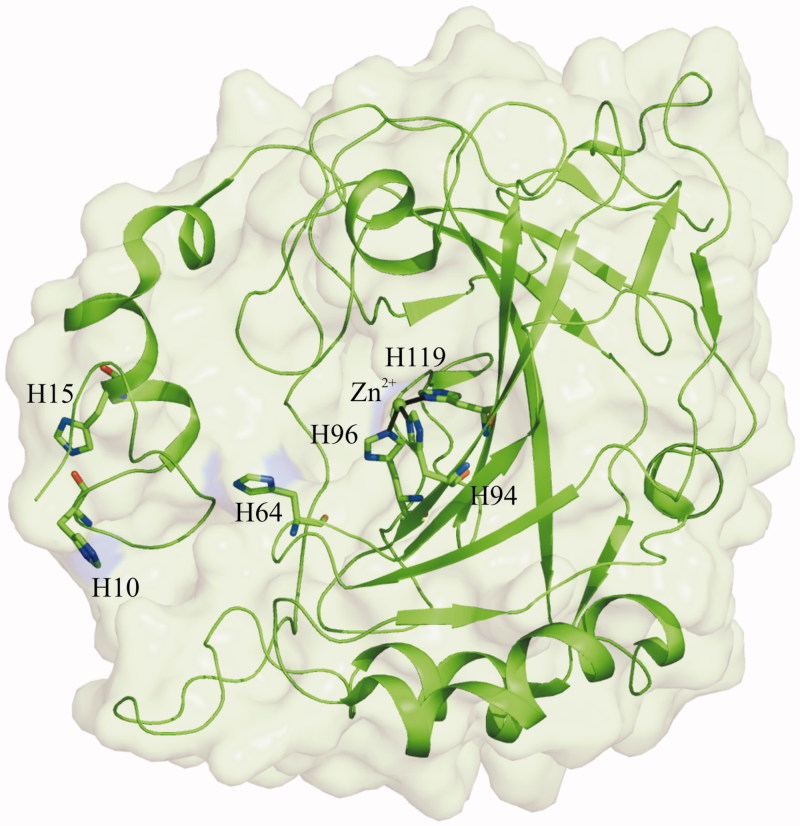
hCA XIII structure (PDB accession code 3D0N). His10, His15, His64, and zinc-coordinating histidines are shown in stick representation.

In order to design suitable hCA XIII variants, a careful comparison between the crystallographic structures of hCA XIII^18^ and hCA II^10^ was carried out. In agreement with their high sequence identity, these two isoforms have a high degree of three-dimensional similarity (Figure 2(A)), with a rmsd value calculated by the superposition of the corresponding Cα atoms of only 0.65 Å. Since the main differences between the two enzymes are located on loop regions of the protein surface far from the active site^18^, these cannot be considered as responsible for their different catalytic features. Focusing the attention to the active site region, it is evident that most of the residues delimiting this cavity are generally conserved by nature and conformation. Only six residues within hCA XIII active site cavity differ from those of hCA II, namely Ser62, Ser65, Arg91, Val132, Ala135, and Val200 that are substituted by Asn, Ala, Ile, Gly, Val, and Thr, respectively (Figure 2(B)). Single-site mutations were then carried out on hCA XIII residues in position 62, 65, and 200 which have been reported in hCA II to be involved in the stabilisation of the solvent network within the active site (see introduction)^35^^,^^36^^,^^47^ and on residue in position 91, due to its neighbourhood to the substrate binding pocket defined by residues Val121, Val143, Leu198, and Trp209. On the contrary, the inspection of hCA II and XIII structures suggested that the residues in position 132 and 135 were too distant from any molecular mechanism at the basis of the CA activity, thus these positions were disregarded from our investigation. Finally, we aimed to investigate whether the histidine cluster has a role in modulating hCA XIII activity. Indeed, the analysis of hCA XIII structure showed the absence of a well-defined histidine cluster due to the missing of His3 and His4 on the rim of the protein. This absence makes His64 too distant from the external residues His10 and His15, thus not allowing the formation of the proper channel able to connect the active site to the protein surface (Figure 3). To verify this hypothesis^18^^,^^38^, an additional hCA XIII variant, where residues Leu3 and Ser4 were substituted with histidines, was designed. In total, five hCA XIII mutants, namely L3H/S4H, S62N, S65A, R91I, and V200T were designed, cloned, expressed, and purified with a homogeneity degree above 98%. Correctness of single-site mutations was confirmed by DNA sequencing, whereas LC-ESI-MS experiments confirmed the integrity and purity of the recombinant proteins. CO_2_ hydration activity of the purified mutants was determined by a stopped-flow CO_2_ hydration assay and compared with activity of the wild-type hCA XIII. Results, reported in Table 1, show that most of hCA XIII variants were affected by replacement(s). In particular, V200T resulted the most active variant with a significant increase of 67% of the catalytic efficiency. The contribution of S65A and S62N to the overall catalytic efficiency was also evident with an improvement of 48% and 30%, respectively. Interestingly, also the variant L3H/S4H showed a rather good improvement of the hCA XIII catalytic efficiency (about 22%), supporting the debated role of the histidine cluster in modulating the proton transfer reaction^18^. It is worth noting that, despite the different polarity feature, R91I replacement did not affect CA activity, thus indicating that residue in position 91 is not essential to CA catalysis, in agreement with the observation that it is highly variable among the 12 catalytically active isoforms.

**Table 1.** Catalytic features of wild type hCA XIII, its five variants and for comparison hCA II.

Isoform	Mutation	*k*_cat_ (s^–1^)	*K*_M_ (mM)	*k*_cat_/*K*_M_ (M^–1^ s^–1^)	*K*_I_ (AZM) (nM)
hCA XIII	wt	1.5 × 10^5^	13.8	1.08 × 10^7^	16
hCA XIII	L3H/S4H	1.8 × 10^5^	13.6	1.32 × 10^7^	11
hCA XIII	S62N	1.4 × 10^5^	10.1	1.40 × 10^7^	105
hCA XIII	S65A	1.6 × 10^5^	10.0	1.60 × 10^7^	23
hCA XIII	R91I	1.6 × 10^5^	14.5	1.10 × 10^7^	108
hCA XIII	V200T	1.7 × 10^5^	9.4	1.80 × 10^7^	13
hCA II	wt	1.4 × 10^6^	9.3	1.50 × 10^8^	12

Inhibition data with acetazolamide (AZM), a well-known CA inhibitor, are also reported.

## Conclusions

The aim of this work was to identify hCA XIII residues that determine the lower catalytic efficiency of this isoform with respect to the most efficient hCA II. Upon structural comparison, diverging residues involved in the formation of an ordered water network within the catalytic site, of a histidine cluster on the protein surface, or located in the proximity of the substrate binding pocket were replaced with those corresponding to hCA II by a site-specific mutagenesis approach. Although none of the designed variants fully restored hCA II activity, which is driven by a multifactorial basis, most of the single mutations were able to improve the enzymatic activity. Overall, our results add another piece in the puzzle of hCA catalytic mechanism highlighting that residues assisting the formation of the ordered solvent network are important to guarantee an efficient proton transfer. Moreover, also the histidine cluster represents a structural feature that contributes to the catalytic rate of this superfamily of enzymes.
